# The oral administration of *Lacticaseibacillus casei* Shirota alleviates acetaminophen-induced liver injury through accelerated acetaminophen metabolism via the liver-gut axis in mice

**DOI:** 10.1128/msphere.00672-23

**Published:** 2024-01-09

**Authors:** Longxian Lv, Siqi Ren, Huiyong Jiang, Ren Yan, Wenyi Chen, Ruiyi Yan, Jinming Dong, Li Shao, Ying Yu

**Affiliations:** 1 State Key Laboratory for Diagnosis and Treatment of Infectious Diseases, Collaborative Innovation Center for Diagnosis and Treatment of Infectious Diseases, The First Affiliated Hospital, College of Medicine, Zhejiang University, Hangzhou, Zhejiang, China; 2 Jinan Microecological Biomedicine Shandong Laboratory, Jinan, Shandong, China; 3 Key Laboratory of Biomarkers and In Vitro Diagnosis Translation of Zhejiang Province, School of Public Health, Hangzhou Medical College, Hangzhou, Zhejiang, China; 4 School of Clinical Medicine, Hangzhou Medical College, Hangzhou, Zhejiang, China; 5 The Affiliated Hospital of Hangzhou Normal University, Institute of Translational Medicine, Hangzhou Normal University, Hangzhou, Zhejiang, China; University of California, Davis, Davis, California, USA

**Keywords:** liver injury, acetaminophen, *Lacticaseibacillus casei *Shirota, liver-gut axis, glutathione

## Abstract

Acetaminophen is a widely used antipyretic and analgesic drug, and its overdose is the leading cause of drug-induced acute liver failure. This study aimed to investigate the effect and mechanism of *Lacticaseibacillus casei* Shirota (LcS), an extensively used and highly studied probiotic, on acetaminophen-induced acute liver injury. C57BL/6 mice were gavaged with LcS suspension or saline once daily for 7 days before acute liver injury was induced via intraperitoneal injection of 300 mg/kg acetaminophen. The results showed that LcS significantly decreased acetaminophen-induced liver and ileum injury, as demonstrated by reductions in the increases in aspartate aminotransferase, total bile acids, total bilirubin, indirect bilirubin, and hepatic cell necrosis. Moreover, LcS alleviated acetaminophen-induced intestinal mucosal permeability, decreased serum IL-1α and lipopolysaccharide levels, and elevated serum eosinophil chemokine (eotaxin) and hepatic glutathione levels. Furthermore, analysis of the gut microbiota and metabolome showed that LcS reduced the acetaminophen-enriched levels of *Cyanobacteria*, *Oxyphotobacteria*, long-chain fatty acids, cholesterol, and sugars in the gut. Additionally, the transcriptomic and proteomic results showed that LcS mitigated the decrease in metabolic and immune pathways as well as glutathione formation during acetaminophen-induced acute liver injury. This is the first study showing that pretreatment with LcS alleviates acetaminophen-enriched acute liver injury, and it provides a reference for the application of LcS.

**IMPORTANCE:**

Acetaminophen is the most frequently used antipyretic analgesic worldwide. As a result, overdoses easily occur and lead to drug-induced acute liver injury, which quickly progresses to liver failure with a mortality of 60%–80% if not corrected in time. The current emergency treatment for overused acetaminophen needs to be administered within 8 hours to avoid liver injury or even liver failure. Therefore, developing preventive strategies for liver injury during planned acetaminophen medication is particularly important, preferably nonpharmacological methods. *Lacticaseibacillus casei* Shirota (LcS) is a famous probiotic that has been used for many years. Our study found that LcS significantly alleviated acetaminophen-induced acute liver injury, especially acetaminophen-induced liver injury toward fulminant hepatic failure. Here, we elucidated the function and potential mechanisms of LcS in alleviating acetaminophen-induced acute liver injury, hoping it will provide preventive strategies to people during acetaminophen treatment.

## INTRODUCTION

Acetaminophen/paracetamol (APAP) is the most widely used antipyretic analgesic worldwide (1). This drug is recommended at 650–1,000 mg every 4–6 hours for adults and no more than 4 g every day. Under this dosing scheme, most APAP can be detoxified via glucuronidation and sulfation and then excreted in the urine. The remaining APAP is converted to hepatotoxic N-acetyl-para-benzoquinone amine (NAPQI) by cytochrome P450; this molecule is reduced by glutathione to nontoxic and soluble mercapturic acid and excreted in the urine. However, due to the ubiquity and wide availability of this drug, the intentional or unintentional overdose of APAP often occurs, and in these cases, excessive NAPQI may cause acute liver injury (ALI) after the exhaustion of glutathione (2). This is the most common type of drug-induced liver injury, which is responsible for 40%–70% of ALI in the United States and Europe (2). In China, this proportion has reached 20.7% (3). N-acetyl cysteine is an antidote for excessive APAP, which requires timely detoxification within 8 hours (4). However, people often miss the optimal detoxification time because APAP overdose usually occurs unconsciously. If not corrected in time, ALI can further develop into life-threatening hepatic failure and exhibit mortality rates as high as 60%–80% (5).

Recent studies have shown that the gut microbiota contributes greatly to APAP-induced ALI. Schneider et al. found that intestinal dysbiosis is transferrable to healthy wild-type mice via fecal microbiota transfer and aggravated liver injury, which indicated that the gut microbiota is a targetable risk factor (6). Zheng et al. found that pretreatment with vancomycin to deplete the gut microbiota attenuates APAP-induced liver injury through 2-hydroxybutyric acid (7), which further confirms the critical role of the gut microbiota during this process. Using APAP-induced ALI, Kolodziejczyk et al. found that acute liver failure is regulated by the *Myc* gene and gut microbiota (8), which partially explains the mechanism through which the gut microbiota participates in APAP-induced ALI. To date, a few probiotics have been reported to alleviate APAP-induced ALI in mice. such as *Lactobacillus acidophilus* LA14 and *Limosilactobacillus reuteri* DSM 17938. However, few of them have conducted in-depth research on the mechanism (9, 10).

*Lacticaseibacillus casei* Shirota (LcS) is one of the most studied industrially produced lactic acid bacteria in the world, and its functions include regulation of gut microbiology (11), the immune system (12), relief of neurological diseases (13, 14), and maintenance of lung, stomach, and intestinal health (14–16). LcS has also shown promising performance in alleviating liver disease. Wagnerberger et al. (17) found that the dietary intake of LcS can prevent fructose-induced nonalcoholic fatty liver disease by reducing the hepatic TLR-4 signaling cascade. Yan et al. (11) found that LcS reduces excessive inflammation and metabolic disorders by reshaping the gut microbiota to alleviate D-galactosamine-induced ALI. Kumar et al. (18) and Nikbakht Nasrabadi et al. (19) found that LcS reduces the serum aflatoxin levels in aflatoxin-treated rats to lower the risk of liver cancer. However, whether LcS can alleviate ALI caused by APAP has not been reported.

In this study, the effect of LcS on APAP-induced ALI was investigated using a mouse model. The inflammatory cytokines, gut microbiota, metabolome, transcriptome, and proteomics were assayed to explore the underlying mechanism.

## RESULTS

### Pretreatment with LcS alleviates APAP-induced ALI and the partly abnormal release of cytokines

**Fig 1:**
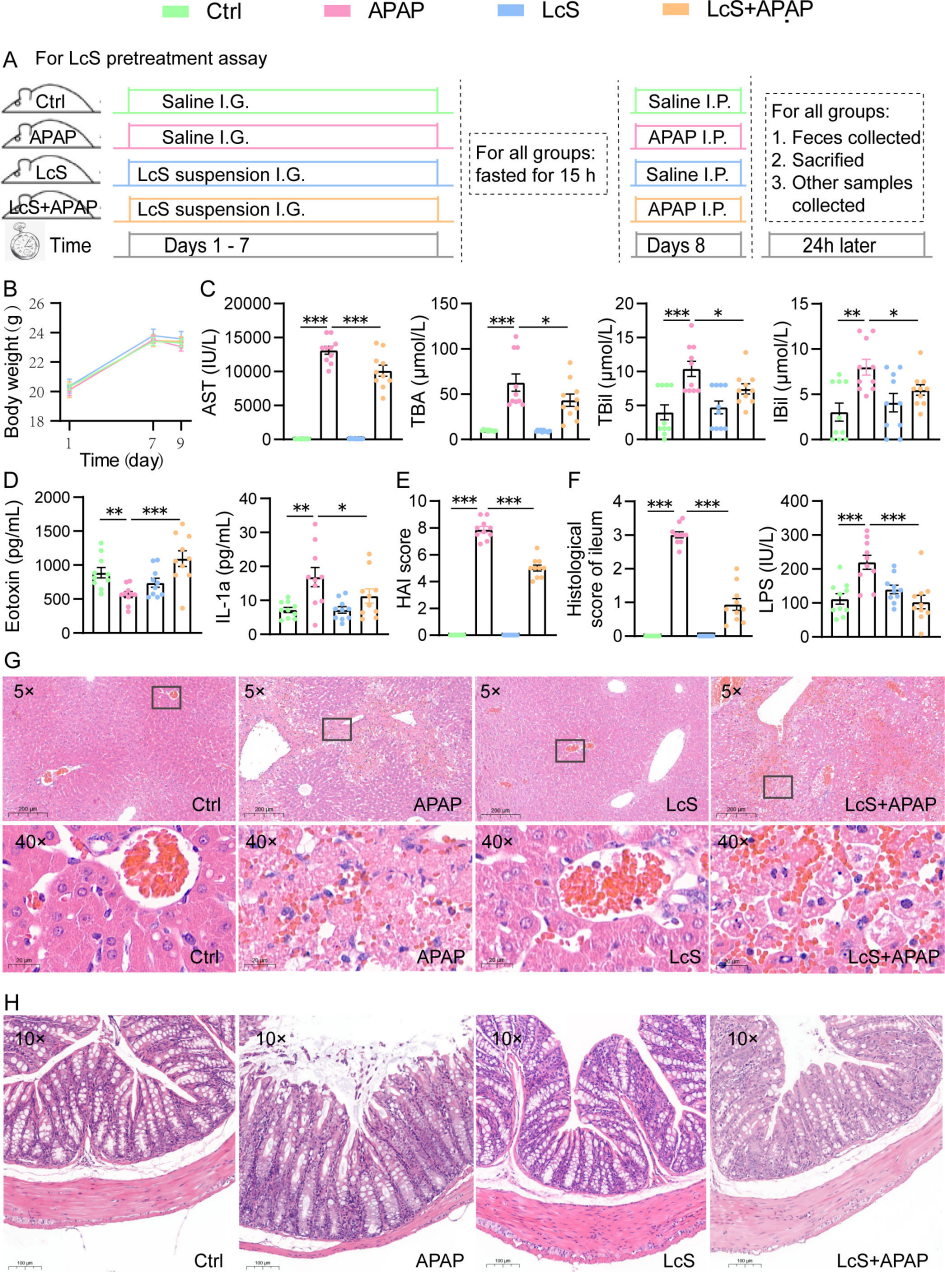
LcS alleviates APAP-induced ALI and gut injury. (**A**) Animal experimental procedure of the LcS pretreatment assay. (**B**) Body weight of mice during the experiment. LcS decreased the APAP-induced (**C**) increases in liver function indicators, including AST, TBA, TBil, and IBil. (**D**) Elevations in the serum cytokine eotaxin and decline in IL-1α. (**E**) Increases in the hepatic histological activity index (HAI) score. (**F**) Increases in the histological scores of the ileum and serum lipopolysaccharide (LPS). (**G**) Representative images of hepatic hematoxylin-eosin (HE) staining; the scale bars represent 200 µm and 20 µm at 5× and 40× magnification, respectively. (**H**) Representative images of ileal HE staining; the scale bar represents 100 µm. The data are shown as the means ± SEMs; *P < 0.05, **P < 0.01, and ***P < 0.001.

Twenty-four hours after APAP administration, no mice died, and no significant difference in body weight was observed among the groups (Fig. 1B). An analysis of liver function revealed that APAP significantly increased the serum levels of alanine aminotransferase (ALT), aspartate aminotransferase (AST), glycyl proline dipeptidyl aminopeptidase (GPDA), alkaline phosphatase, total bile acid (TBA), total bilirubin (TBil), direct bilirubin (DBil), and indirect bilirubin (IBil) and decreased the total protein and cholinesterase levels (Table S2). LcS decreased the APAP-induced increases in AST, TBA, TBil, and IBil but did not alter any liver function indicators in healthy mice (Fig. 1C).

A hepatic histological examination showed that the APAP-exposed liver exhibited marked piecemeal necrosis (portal vein with more than 50% of its circumference) and few erythrocytes in vessels at 5× magnification. Further magnification (40×) revealed that the necrotic foci presented multilobular necrosis, disappearance of nuclei, and infiltration of erythrocytes and exhibited a hepatic histological activity index (HAI) (20) of 7.88 (Fig. 1E and G). LcS decreased multilobular necrosis and nuclear disappearance and alleviated liver injury to the level of moderate piecemeal necrosis (portal vein circumference less than 50%, HAI = 5 points). In addition, LcS had no significant effect on liver histology in healthy mice (Fig. 1E and G).

In mice with APAP-induced ALI, the serum levels of the cytokines eotaxin, MIP-1α, IL-1β, IL-3, IL-12p70, IL-13, IL-17α, IFN-γ, and TNF-α were decreased, whereas the levels of G-CSF, MCP-1, CXCL-1, IL-1α, and IL-6 were elevated (Table S3). LcS reduced the APAP-induced decrease in eotaxin and increased the IL-1α levels (Fig. 1D).

### Pretreatment with LcS reduces APAP-induced intestinal permeability and intestinal mucosal injury

Hematoxylin-eosin (HE) staining of the ileum revealed that APAP caused necrosis of epithelial cells at the top of intestinal villi and cracking of many intestinal villi, which is the primary histological feature of intestinal mucosal injury. The ileum histological score was 3 (Fig. 1F and H). Furthermore, lipopolysaccharide (LPS), which is both an inducer and an indicator of increased intestinal mucosa permeability, was increased in the serum of APAP-exposed mice (Fig. 1F). Pretreatment with LcS significantly reduced the LPS increase and gut villus cracking induced by APAP and decreased the ileum histological score to 0.94. In addition, LcS did not damage the intestinal mucosa of healthy mice. These results suggest that LcS alleviates APAP-induced intestinal permeability and intestinal mucosal injury.

### Pretreatment with LcS reduced APAP-induced alterations of gut microbiota

**Fig 2:**
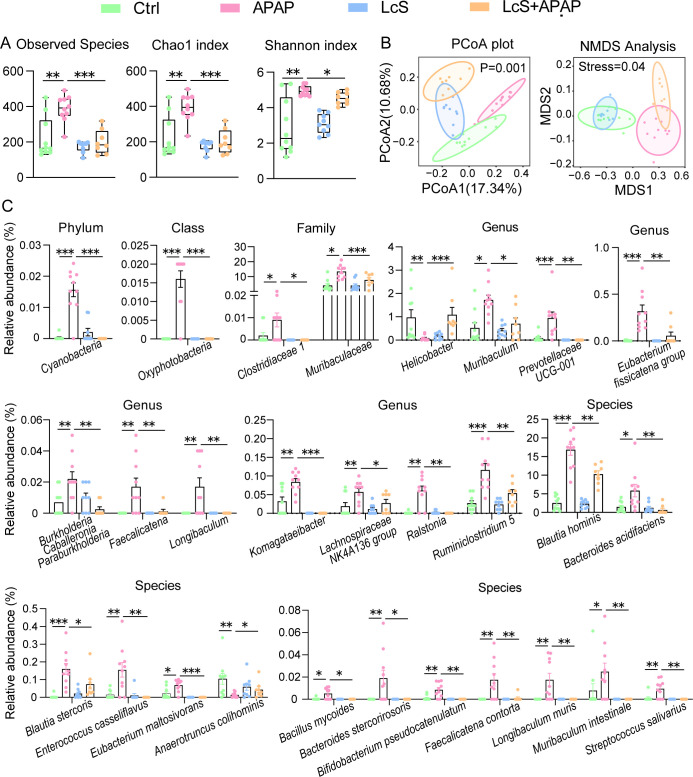
LcS alleviates APAP-induced gut microbiota dysbiosis. (**A**) Differences in α-diversity: observed species, Chao1 and Shannon indices. The values are expressed as the medians with interquartile ranges. (**B**) Differences in β-diversity determined by PCoA and NMDS. (**C**) LcS alleviated some APAP-induced alterations in the gut microbiota. The data are shown as the means ± SEMs; *P < 0.05, **P < 0.01, and ***P < 0.001.

LcS significantly reduced the APAP-induced increase in the α-diversity of the gut microbiota, including the observed species, Chao1 index and Shannon index, which reflect the species number, richness, and evenness of the gut microbiota, respectively (Fig. 2A). The β-diversity represents differences in the microbial community structure, and the unweighted UniFrac method based on principal coordinates analysis (PCoA) showed that each group was well differentiated from each other. Permutational multivariate analysis of variance (*P* = 0.001) and multidimensional scaling (NMDS, stress value = 0.04) also indicated that the microbiota of each group exhibited significant differences in structure (Fig. 2B).

The improvement of APAP-induced gut microbiota dysbiosis by LcS was mainly reflected in its prevention of microbial enrichment (Fig. 2C). These microbes include *Cyanobacteria* at the phylum level; *Oxyphotobacteria* at the class level; *Clostridiaceae* 1 and *Muribaculaceae* at the family level; *Muribaculum*, *Prevotellaceae* UCG-001, *Eubacterium fissicatena* group, *Burkholderia Caballeronia Paraburkholderia*, *Faecalicatena*, *Longibaculum*, *Komagataeibacter*, *Lachnospiraceae* NK4A136 group, *Ralstonia*, and *Ruminiclostridium* 5 at the genus level; and *Blautia hominis*, *Bacteroides acidifaciens*, *Blautia stercoris*, *Enterococcus casseliflavus*, *Eubacterium maltosivorans*, *Bacillus mycoides*, *Bacteroides stercorirosoris*, *Bifidobacterium pseudocatenulatum*, *Faecalicatena contorta*, *Longibaculum muris*, *Muribaculum intestinale*, and *Streptococcus salivarius* at the species level. In addition, LcS also reduced the APAP-induced depletion of *Helicobacter* at the genus level and *Anaerotruncus colihominis* at the species level.

### Pretreatment with LcS improves APAP-induced gut metabolic disorders

**Fig 3:**
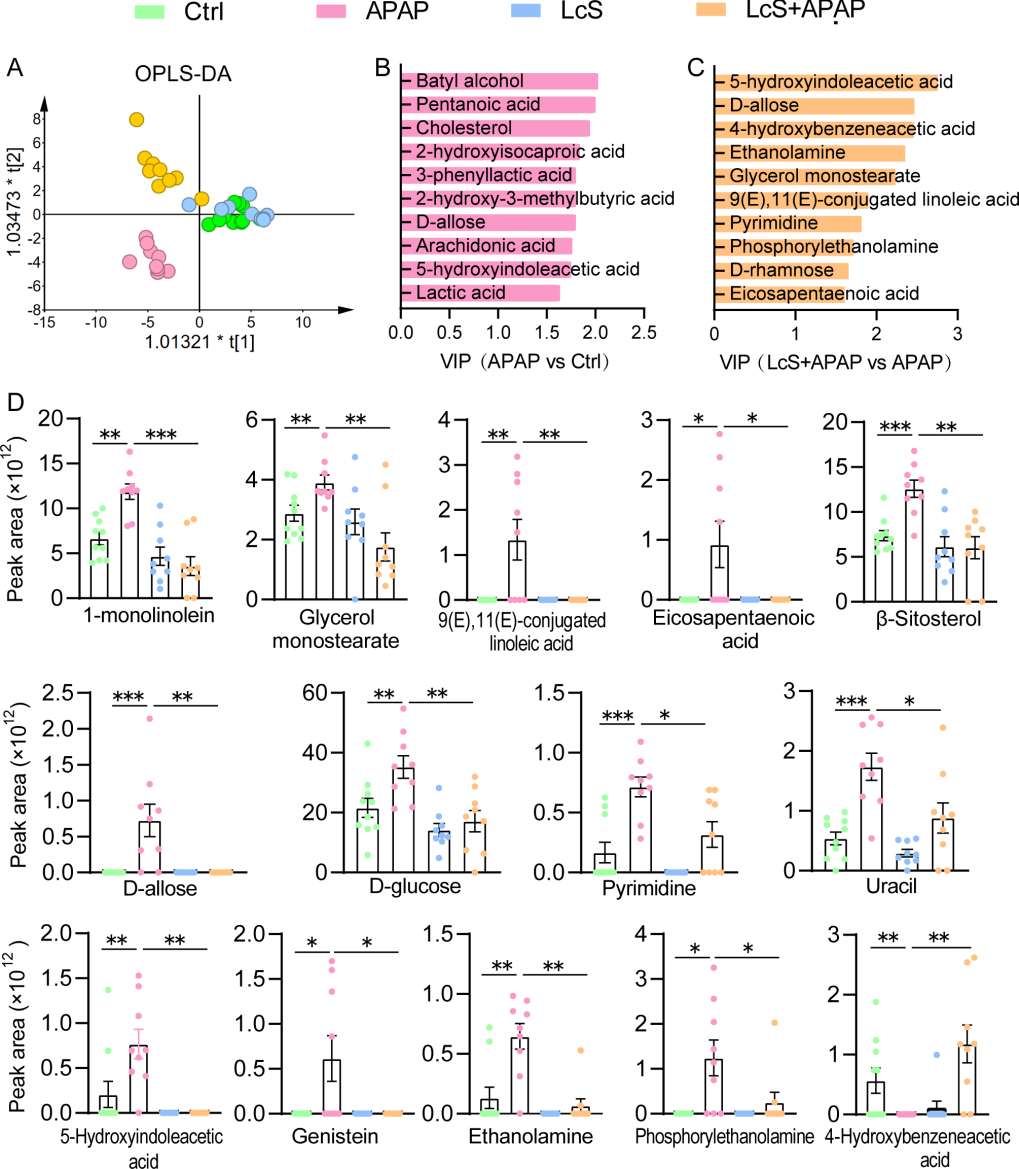
LcS alleviates APAP-induced gut metabolome dysbiosis. (**A**) OPLS-DA score plots of metabolome profiles. (**B**) Top 10 metabolites contributing to the separation of the APAP group from the Ctrl group based on VIP values. (**C**) Top 10 metabolites contributing to the separation of the LcS + APAP group from the APAP group based on VIP values. (**D**) LcS alleviated some APAP-induced alterations in gut metabolites. The data are shown as the means ± SEMs; *P < 0.05, **P < 0.01, and ***P < 0.001.

GC–MS analysis identified a total of 106 metabolites in feces, including fatty acids, esters, amino acids, sugars, and their derivatives. Orthogonal partial least squares discriminant analysis (OPLS-DA) showed that these four groups were separated, indicating that their metabolomes were quite different (Fig. 3A). The variable importance in the projection (VIP) analysis showed that the top 10 metabolites contributing to the distinction between the APAP group and the Ctrl group were batyl alcohol, pentanoic acid, cholesterol, 2-hydroxyisocaproic acid, 3-phenyllactic acid, 2-hydroxy-3-methylbutyric acid, d-allose, arachidonic acid, 5-hydroxyindoleacetic acid, and lactic acid (Fig. 3B). 5-Hydroxyindoleacetic acid, d-allose, 4-hydroxybenzeneacetic acid, ethanolamine, glycerol monostearate, 9(E),11(E)-conjugated linoleic acid, pyrimidine, phosphorylethanolamine, d-rhamnose, and eicosapentaenoic acid were the top 10 metabolites contributing to the difference between the LcS + APAP group and the APAP group (Fig. 3A and C).

Pretreatment with LcS significantly reduced the APAP-induced enrichment of 13 metabolites, including two long-chain fatty acids [9(E),11(E)-combined linoleic acid and eicosapentaenoic acid], two esters (1-monoolein and glycol monostearate), one sterol (β-sitosterol), two sugars (d-allose and d-glucose), two nucleic acids (pyrimidine and uracil), and four other chemicals (5-hydroxyindoleacetic acid, genistein, ethanolamine, and phosphorylethanolamine). Moreover, pretreatment with LcS significantly ameliorated the APAP-induced depletion of 4-hydroxybenzeneacetic acid (Fig. 3D).

### Pretreatment with LcS mainly improves the APAP-induced downregulation of the liver metabolic pathway

**Fig 4:**
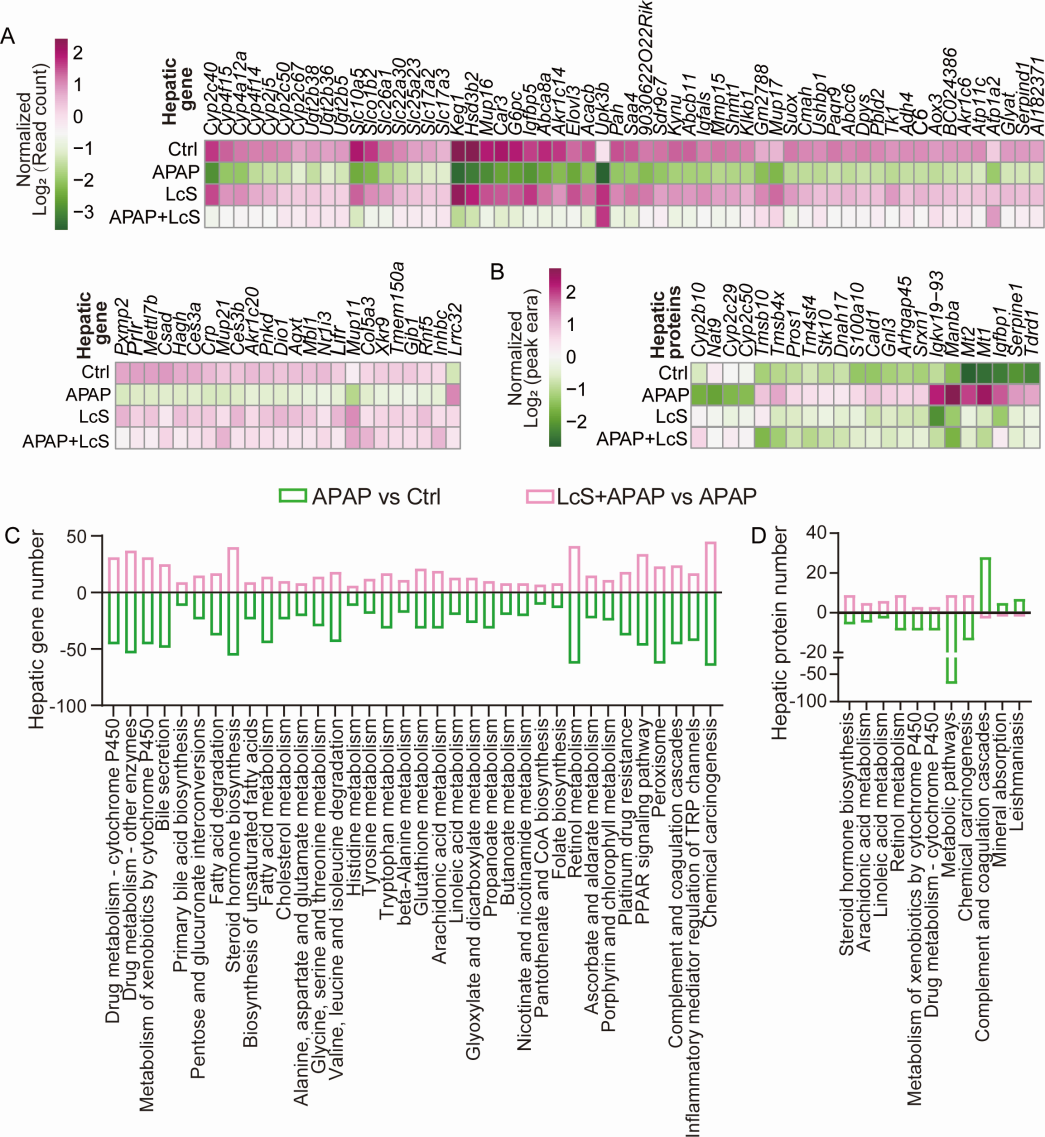
LcS alleviates APAP-induced changes in the liver transcriptome and proteome. LcS alleviates the APAP-induced alterations in gene transcription (**A**) and protein expression (**B**) in the liver. All these data were normalized to those of the Ctrl group. LcS alleviates APAP-induced pathway alterations in the transcriptome (**C**) and proteome (**D**) of the liver.

The liver transcriptome showed that LcS alleviated the changes in 1,058 genes altered by APAP at a significance level of *P* < 0.05, and 82 of these genes met the criteria of *P*_adj_ < 0.05 and |log2 (fold change)| ≥ 1 (Fig. 4A). First, LcS mitigated the APAP-induced transcript downregulation of liver genes in known pathways of APAP metabolism. These genes include glutathione S-transferase genes (*Gstt1*, *Gstt2*, *Gstt3*, *Gstm1*, *Gstm2*, *Gstm2-ps1*, *Gstm4*, *Gstm6*, *Gsta3*, *Gstk1*, *Gstp1*, and *Mgst1*), aminopeptidase N gene (*Anpep*), and N-acetyltransferase genes (*Nat8f2* and *Nat8f1*), which are necessary for the metabolism of NAPQI into nontoxic mercapturic acid via glutathione; the *Idh1* gene, which converts NADP+ to NADPH and thereby reduces glutathione disulfide to glutathione (Fig. 6A); some genes in the UDP (uridine diphosphate) -glucuronosyltransferase family (*Ugt2b5*, *Ugt2b34*, and *Ugt2b36*) and cytochrome P450 family genes (such as *Cyp2c40*, *Cyp2c50*, and *Cyp4f15*). Second, LcS mitigated the APAP-induced downregulation of genes related to synthesis, metabolism, and transportation, such as genes related to the synthesis and metabolism of carbohydrates (*Keg1* and *G6pc*), proteins (*Pah*, *Shmt1*, and *Glyat*), and fats (*Hsd3b2* and *Acacb*), as well as sodium-dependent phosphate transport genes (*Slc10a5*, *Slc26a1*, and *Slc17a3*). Moreover, LcS also mitigated the APAP-induced downregulation of genes related to immunity (*C6* and *Crp*) and antioxidation (*Pxmp2*, *Suox*, and *Hagh*).

KEGG pathway enrichment analysis of the transcriptome showed that pretreatment with LcS improved the APAP-induced downregulation of 36 pathways in the liver (Fig. 4C, *P*_adj_ < 0.05). On one hand, most of these pathways were related to metabolism. First, LcS improved the APAP-induced downregulation of pathways related to xenobiotic biodegradation and metabolism, including the metabolism of xenobiotics by cytochrome P450, drug metabolism and other enzymes. Second, LcS improved the APAP-induced downregulation of lipid metabolism pathways, including primary bile acid biosynthesis, fatty acid degradation, fatty acid metabolism, biosynthesis of unsaturated fatty acids, steroid hormone biosynthesis, arachidonic acid metabolism, and linoleic acid metabolism pathways. Third, LcS improved the APAP-induced downregulation of carbohydrate metabolism pathways, including glyoxylate and dicarboxylate metabolism, pentose and glucuronate interconversions, propanoate metabolism, butanoate metabolism, and ascorbate and aldarate metabolism pathways. Fourth, LcS improved the APAP-induced downregulation of pathways of amino acid metabolism, including alanine, aspartate, and glucose metabolism; glycine, serine, and threonine metabolism; valine, leucine, and isoleucine degradation; histidine metabolism; tyrosine metabolism; tryptophan metabolism; beta-alanine metabolism; and glutathione metabolism. Moreover, LcS improved the APAP-induced downregulation of pathways of metabolism of cofactors and vitamins, including the nicotinate and nicotinamide metabolism, pantothenate and CoA biosynthesis, folate biosynthesis, retinol metabolism, and porphyrin and chlorophyll metabolism pathways. On the other hand, pretreatment with LcS improved the APAP-induced downregulation of pathways related to organic systems, including the digestive system (bile secretion and cholesterol metabolism), endocrine system (PPAR signaling pathway), immune system (complement and coagulation cascades), and sensor system (inflammatory mediator regulation of TRP channels). In addition, LcS also improved the downregulation of cancer (chemical carcinogenesis) and drug resistance (platinum drug resistance) signaling pathways and transport and catabolism (peroxisome)-related signaling pathways in human diseases.

Proteome analysis was conducted to further explore the mechanism by which LcS pretreatment improves APAP-induced ALI. LcS alleviated the APAP-induced decrease in four proteins (Fig. 4B), including N-acetyltransferase (Nat9) and cytochrome P450 family proteins (Cyp2c29, Cyp2c50, and Cyp2b10). LcS also alleviated the APAP-induced increase in 18 proteins (Fig. 4B); among these proteins, fifteen have been reported to stimulate liver diseases or tumor formation, including Manba, Stk10, Tmsb10, Cald1, Serpine1, Mt1, Mt2, Igfbp1, Tm4sf4, Pros1, S100a10, Dnah17, Gnl3, Tdrd1, and Srxn1; the remaining three are Igkv19-93, Arhgap45, and Tmsb4x, which are related to the formation of antibodies and the activation of immune cells. The KEGG pathway analysis using these proteome results showed that pretreatment with LcS alleviated the APAP-induced downregulation of eight pathways at the protein level (Fig. 4D), and most of these pathways were the same as those at the transcript level, including pathways of steroid hormone biosynthesis, arachidonic acid metabolism, linoleic acid metabolism, retinol metabolism, metabolism of xenobiotics by cytochrome P450, drug metabolism-cytochrome P450, and chemical carcinogenesis. In addition, LcS alleviated the APAP-induced upregulation of three pathways, including complement and coagulation cascades, mineral absorption, and leishmaniasis.

### Pretreatment with LcS improves the APAP-induced inhibition of intestinal pathways related to mucosal immunodeficiency

**Fig 5:**
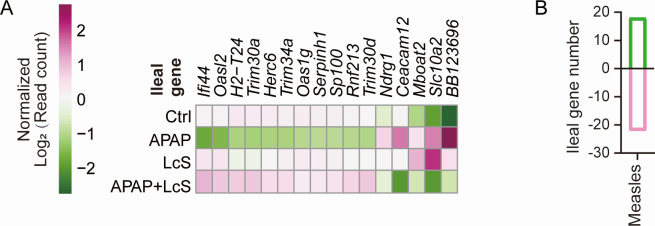
LcS alleviates the APAP-induced alterations in gene transcription (**A**) and pathways (**B**) in the terminal ileum. The gene transcription data were normalized to those of the Ctrl group.

The ileal transcriptome showed that LcS reduced the APAP-induced upregulation of five genes [*P*_adj_ <0.05, |log2 (fold change)| ≥ 1], including *NDRG1* and *ceacam12*, which are related to cancer; *Slc10a2*, which transports intestinal bile; and *Mboat2* and *BB123696*, which have unclear functions. LcS improved the APAP-induced downregulation of 11 genes: *Ifi44*, *H2-T24*, *Oasl2*, *Oas1g*, *Trim30a*, *Trim34a*, and *Trim30d*, which are needed to maintain normal immune function; *Serpinh1*, *Sp100*, and *Rnf213*, which regulate tumors; and *Herc6*, which has an uncertain function (Fig. 5A). In addition, a KEGG analysis showed that LcS improved APAP-induced downregulation of the measles pathway (Fig. 5B).

### The conjoint analysis emphasizes the involvement of the glutathione pathway in the alleviation of APAP-induced ALI by LcS

**Fig 6:**
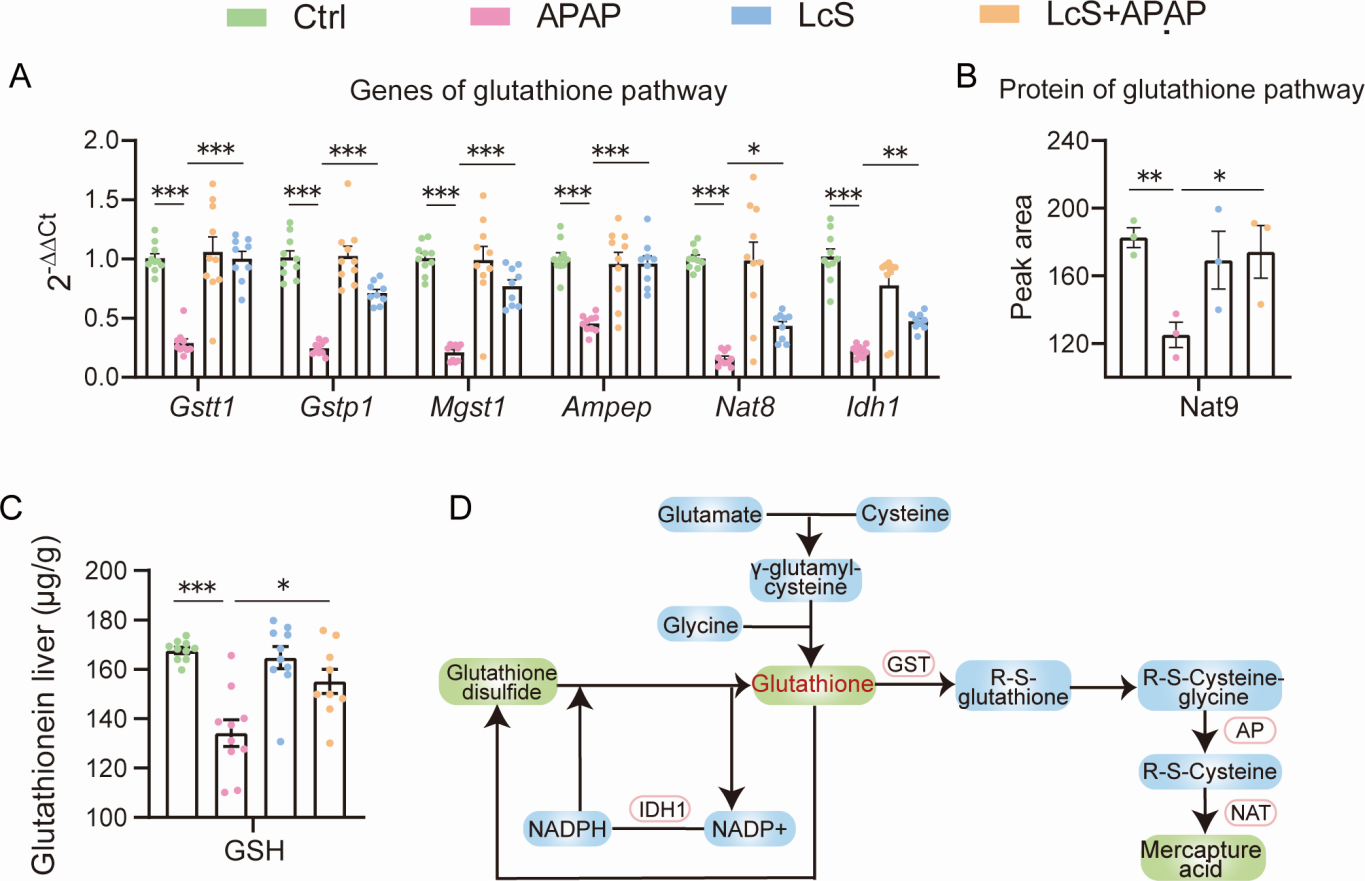
LcS improves the APAP-induced downregulation of the glutathione pathway. (**A**) LcS improves the APAP-induced downregulation of the transcription of genes in the glutathione pathway, as demonstrated by RT-qPCR. (**B**) LcS alleviated the APAP-induced downregulation of the expression of proteins in the glutathione pathway, as exhibited by proteomics. (**C**) LcS increased the APAP-induced decrease in glutathione in the liver, as shown by ELISA. (**D**) General view of the role of LcS in alleviating the APAP-induced downregulation of glutathione-related genes. The data are shown as the means ± SEMs; *P < 0.05, **P < 0.01, and ***P < 0.001.

The transcript alterations of key genes related to the glutathione pathway in the liver transcriptome analysis were first verified by quantitative PCR; these genes included glutathione S-transferase genes (*Gstt1*, *Gstp1*, and *Mgst1*), aminopeptidase N gene (*Anpep*), N-acetyltransferase genes (*Nat8*), and the *Idh1* gene. The alleviation of these downregulations by pretreatment with LcS was also observed by qPCR (Fig. 6A). The proteomic results further revealed that LcS alleviated the APAP-induced downregulation of the N-acetyltransferase Nat9 (Fig. 6B). In addition, the enzyme linked immunosorbent assay (ELISA) results confirmed that pretreatment with LcS elevated the level of glutathione in the liver, which was reduced in mice exposed to APAP (Fig. 6C). In total, these results emphasized that the glutathione pathway was involved in the alleviation mechanism of APAP-induced ALI by pretreatment with LcS (Fig. 6D).

### APAP-altered LcS-alleviated gut microbes and metabolites are strongly correlated with hepatic pathways

**Fig 7:**
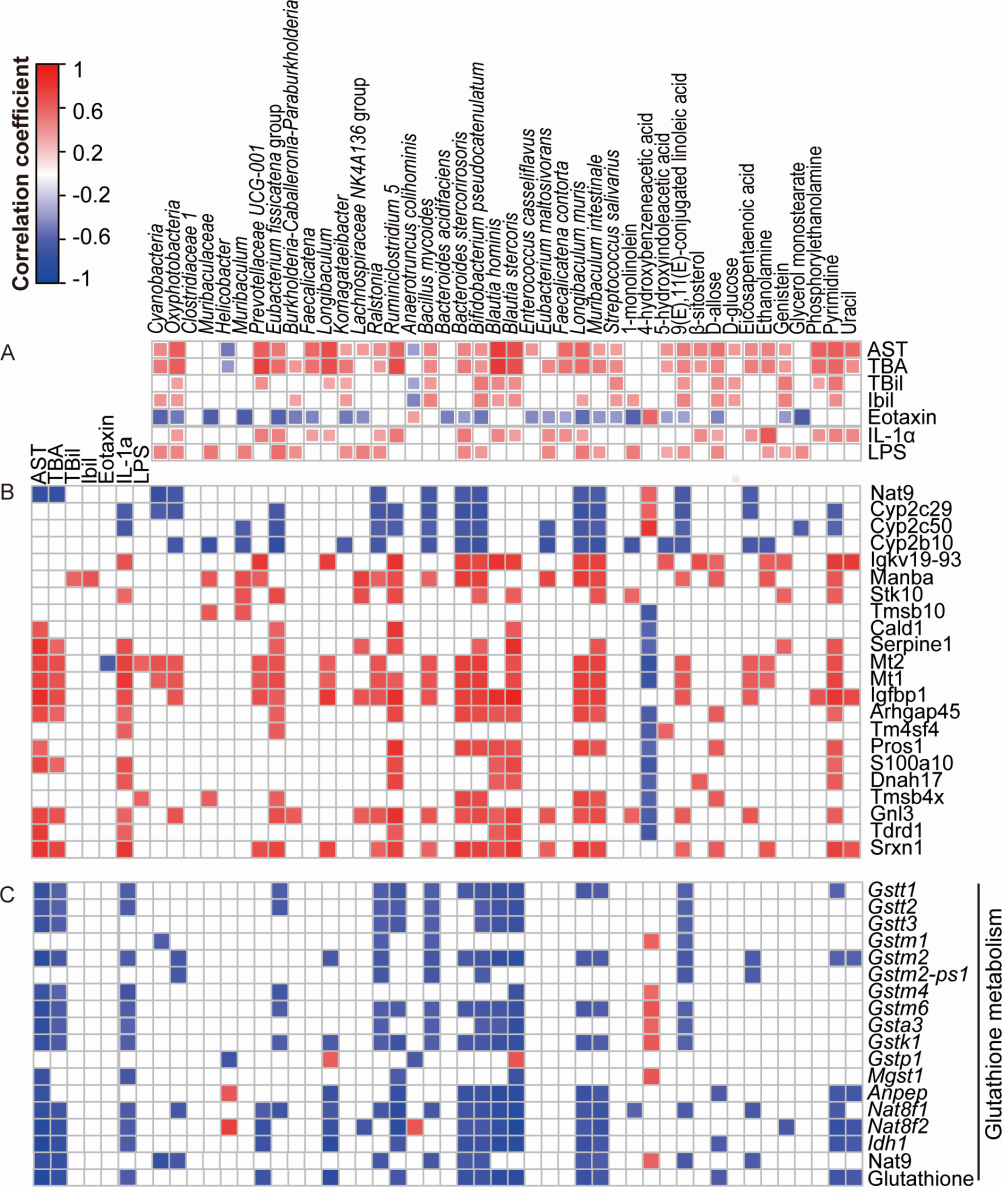
Spearman’s rank correlations between the APAP-induced LcS-alleviated indicators. (**A**) The correlations among the APAP-induced LcS-alleviated gut microbes, metabolites with liver function indicators, inflammatory cytokines, and serum LPS. The correlations among the APAP-induced LcS-alleviated liver function indicators, inflammatory cytokines, serum LPS gut microbes, metabolites with altered proteins (**B**), and indicators in the glutathione metabolism (**C**) of the liver. The results with *P* values < 0.05 are shown in the heatmap. The color key and circle size indicate the strength of the correlation (*r* value). The red color indicates positive correlations; the blue color indicates negative correlations.

The correlations between factors involved in the alleviation of APAP-induced ALI by pretreatment with LcS were analyzed using Spearman’s rank correlation method. First, the relative abundance of most of the APAP-enriched LcS-alleviated gut microbes (such as *Cyanobacteria* and *Oxyphotobacteria*) and gut metabolites [such as 9(E),11(E)-conjugated linoleic acid and d-glucose] was positively correlated with the levels of liver function indicators (AST, TBA, TBil, and IBil), cytokines (such as IL-1α), and LPS (Fig. 7A). Second, the relative abundance of these gut microbiota and gut metabolites, as well as the levels of IL-1a, TBA, and AST, were positively correlated with the transcription and translation of liver genes related to liver diseases and immunity (Fig. 7B; Fig. S1A) but negatively correlated with the transcription and translation of liver genes related to the glutathione metabolism pathway (Fig. 7C). Third, most altered ileum genes were highly correlated with the majority of the altered liver proteins, indicating that close gut-liver communication existed in LcS-mediated ALI alleviation. Specifically, *H2-T24*, *Serpinh1*, *Oas1g*, and *Sp100* were positively correlated with liver proteins, whereas *Ndrg1*, *Mboat2*, *Slc10a2*, and *BB123696* were negatively correlated with liver proteins (Fig. S1).

## DISCUSSION

APAP is the most commonly used antipyretic drug, and despite its clear dosage limits, its overdose is still the leading cause of drug-induced ALI. In addition to emergency treatment after ALI, researchers have also investigated how to prevent and reduce side effects during drug use, such as planned medication after a fever. Therefore, it is important to develop preventive strategies for APAP-induced ALI, preferably nonpharmacological methods. Reportedly, the gut microbiota plays an important role in APAP-induced ALI. Therefore, several probiotics that were readily available for people were screened in our lab, and LcS exhibited significant preventive effects on APAP-induced ALI in animal models.

Male mice were used in our study. One reason is that male animals are more vulnerable because the ovaries of female animals affect the metabolism of glutathione and APAP glucuronidation, which are currently important signaling pathways for studying APAP-induced liver injury (21). Rubin et al. also found that the clinical parameters for APAP-induced ALF, such as AST and bilirubin, in men were much higher than those in women (22). Another reason is that mice are more susceptible than rats to APAP-induced ALI, based on our experience (9).

Serum ALT and AST are the most commonly used indicators of liver injury. Traditionally, patients who overdose APAP go through four clinical stages. The second stage, which is marked by liver injury with elevation of serum ALT and AST, usually begins within 24 hours. The increase in AST after APAP exposure occurs slightly earlier than that of ALT (4). Combined with our results, pretreatment with LcS only alleviated the APAP-induced elevation of AST in serum, which suggests that LcS plays a partial role in the protection of early liver injury in APAP-exposed mice. The bilirubin concentrations usually remain normal initially and may never increase in patients who do not progress to fulminant hepatic failure (4). The LcS-mediated alleviation of the APAP-induced elevation of serum TBil and IBil suggests its potential effect on the prevention of APAP-induced fulminant hepatic failure. An abnormal level of serum bile acid can reflect an imbalance of liver synthesis, secretion, and metabolism and is also an indicator of hepatocyte injury. Wang et al. showed that changes in bile acid signaling can also affect APAP glucuronidation and glutathione regeneration (23). The LcS-mediated alleviation of the APAP-induced increase in serum TBA and the downregulation of primary bile acid biosynthesis and bile secretion in the liver indicated that LcS not only affected the secretion and synthesis of bile in the liver but also affected its circulation in the gut-liver axis. Therefore, pretreatment with LcS alleviates APAP-induced ALI progression toward fulminant hepatic failure.

Our results showed that LcS alleviated the APAP-induced increase in serum IL-1α and decrease in serum eotaxin. Alterations in the levels of serum cytokines are frequently observed in APAP-induced ALI patients (24). This was in line with our observed alterations in the levels of serum cytokines, the transcription of immune-related genes, and immune-related signaling pathways in the APAP-induced ALI mouse model. Among the altered cytokines, it was reported that IL-1α was primarily produced by Kupffer cells, promoted sterile inflammation, and accounted for hepatic injury by a lethal dose of APAP in a mouse model (25). Thus, IL-1α is regarded as a promising therapeutic target for ALI treatment. We found that LcS reduced the APAP-induced increase in serum IL-1α, which, to some extent, proved the effectiveness of LcS in alleviating APAP and indicated its potential working mechanism. In addition, CCL11/eotaxin is an important eosinophil-specific chemokine induced by T helper (Th)−2 cytokines and is associated with the recruitment of eosinophils to sites of inflammation (26). It was reported that eotaxin is upregulated at the transcriptional level during liver regeneration *in vitro* and *in vivo*. Moreover, supplementing eosinophils and eosinophil-derived substances has been proven to have protective effects in many disease models (27). Therefore, the prevention of the APAP-induced decrease in eotaxin further indicates the effectiveness and mechanism of LcS.

LcS was reported to survive passage through the gastrointestinal tract and then regulate the gut microbiota (11, 28), which may contribute greatly to LcS alleviation of APAP-induced ALI. In our study, we found that the α-diversity of the gut microbiota increased in mice exposed to APAP for 24 h, which is in line with a previous report (29). Conversely, Schneider et al. reported that the diversity of gut microbiota was decreased after APAP exposure, which may be due to different APAP dosages and exposure times (6). Furthermore, APAP seems to increase bacteria with the potential to cause diseases. For example, *Cyanobacteria*, a microbe usually found in fresh water, but also detected in the gut of humans and animals, can produce a variety of hepatotoxins (30–32). This microbe was also found to be positively correlated with serum ALI, IL-1α, and the transcription of some genes involved in glutathione metabolism but negatively correlated with serum eotaxin in this study. Therefore, pretreatment with LcS reduced the APAP-induced increase in α-diversity, and alteration of some harmful microbes maintained the overall homeostasis of the gut. In addition, the reduction in the APAP-induced increase in serum LPS by LcS may contribute to the alleviation of ALI because LPS, as a bacterial cell wall component of Gram-negative bacteria, is a main indicator of gut mucosal injury and can provoke severe inflammation and cell death in sepsis, with the liver being the major affected organ (33, 34). Thus, regulation of the gut microbiota may contribute greatly to the improvement effects of LcS on APAP-induced ALI.

Intestinal metabolites are related to digestion, absorption, microbiota, and bile enterohepatic circulation (35). We found that pretreatment with LcS alleviated APAP-induced gut metabolic disorder. On the one hand, pretreatment with LcS alleviated the APAP-induced enrichment of several metabolites that have a potential role in liver injury. For example, the accumulation of conjugated linoleic acid (36), eicosapentaenoic acid (37), and a high concentration of glucose (38) can induce an increase in intestinal permeability and can exacerbate liver injury. Furthermore, conjugated linoleic acid, eicosapentaenoic acid, and β-sitosterol can dissolve in bile, causing biliary tract deposition and liver injury (39–41). In our study, we found that 9(E),11(E)-conjugated linoleic acid and eicosapentaenoic acid were highly negatively correlated with altered genes and proteins in the liver, which suggests that alleviation of the enrichment of these metabolites might be involved in the mechanism through which LcS improves APAP-induced ALI. On the other hand, LcS alleviated the depletion of metabolites that may play a role in the prevention of ALI. For example, 4-hydroxybenzeneacetic acid reportedly has antioxidant potential (42). Therefore, the alleviation of APAP-induced gut metabolic disorder may be one of the important mechanisms of LcS against ALI.

Similar to several studies (9, 43), the mismatching of genes between the transcriptome and proteome was observed in our study, as well as in the glutathione pathway. One reason may be that transcription and translation are two coherent and relatively independent processes that have significantly different half-lives, synthesis rates, and quantities. The other reason is that translation rearranges the mRNA information rather than simply copying the information, resulting in the transcript abundance not being equal to the protein abundance (44).

**Fig 8:**
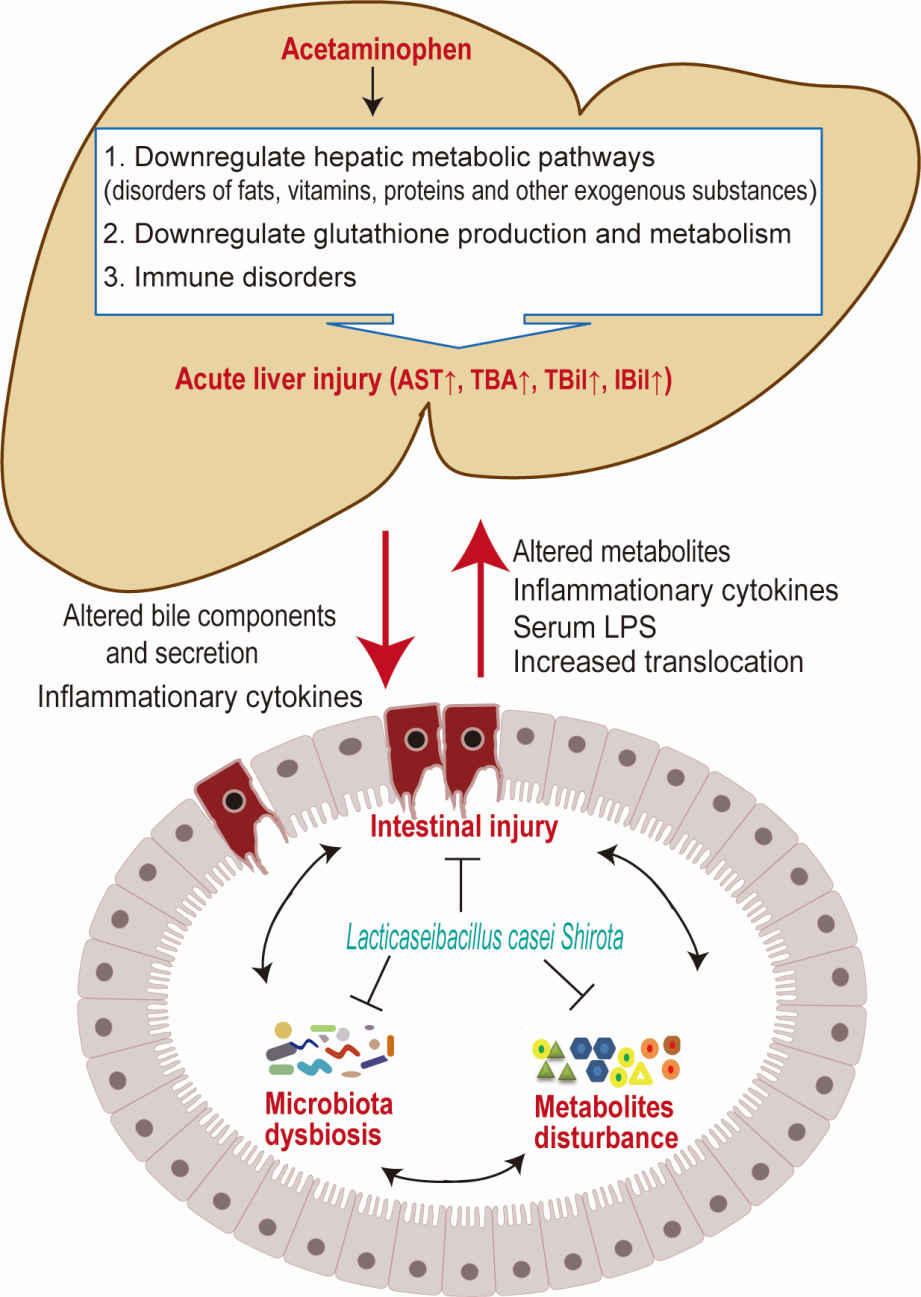
General view of the beneficial effects of LcS on ALI.

The mechanism of APAP-induced ALI is complex, and many intracellular and extracellular events are involved in this pathophysiological process (Fig. 8). LcS alleviated APAP-induced liver injury mainly through the following mechanisms. First, LcS alleviated the downregulation of metabolic pathways, including the pathways of steroid hormone biosynthesis, arachidonic acid metabolism, linoleic acid metabolism, retinol metabolism, metabolism of xenobiotics by cytochrome P450, and drug metabolism-cytochrome P450 at both the transcriptional level and translational level, indicating that LcS alleviated the APAP-induced metabolic disorder of fats, vitamins, proteins, and other exogenous substances, as well as glutathione, which is the pivotal metabolite in mitigating APAP-induced ALI. In addition, LcS partially balanced APAP-induced immune disorders, such as pathways of complement and coagulation cascades, and chemical carcinogenesis, alleviating tumorigenicity, the induction of complement cascades, and abnormal release of some cytokines.

### Conclusion

APAP-induced ALI is characterized by rapid progression, easy neglect in the early stage, and high mortality in the later stage (liver failure). LcS was found to significantly alleviate APAP-induced ALI, especially alleviating APAP-induced ALI progression toward fulminant hepatic failure, through a gut-liver axis involving metabolic pathways and partial immune regulation in our study. We hope that this study will provide a safe, simple, and easily accessible prevention strategy for ALI for people who have to use APAP.

## MATERIALS AND METHODS

### Animal experiment

LcS is derived from Yakult. LcS was anaerobically cultured in de Man, Rogosa and Sharpe (MRS) liquid medium for 24 hours, centrifuged at 8,000 × *g* for 10 min and then suspended in saline (approximately 3 × 10^9^ CFU/mL) to prepare a bacterial suspension.

Male specific-pathogen-free C57BL/6 mice (SLAC, Shanghai, China) were kept under controlled light (12-hour light-dark cycle), temperature (22°C–26°C) and humidity (40%–60%) conditions with free access to food (ZJLAC, Hangzhou, China) and water. For the LcS pretreatment assay, 40 mice that weighed 18–22 g (approximately 8 weeks) were randomly divided into four groups (Fig. 1A). The LcS + APAP and LcS groups were intragastrically injected with 0.2 mL of a suspension of LcS for 7 consecutive days, and the APAP and control groups were both given 0.2 mL of saline instead. On day 8, all the mice were fasted for 15 h; subsequently, an intraperitoneal injection of 300 mg/kg APAP (dissolved in saline at 55°C for 30 min and cooled to room temperature before use; Aladdin, Shanghai, China) was administered to the APAP and LcS + APAP groups, while the same volume of saline was injected into the control and LcS groups. Feces were collected, temporarily stored in liquid nitrogen, and then transferred to −80°C. All animals were then anesthetized with 30 mg/kg pentobarbital sodium and sacrificed 24 hours after APAP administration. Simultaneously, blood was collected from the aorta ventralis and centrifuged at 3,000 × *g* for 10 min to separate the serum, which was used for the detection of liver function and cytokines. Liver (the largest lobe) and ileum specimens (a length of 2 cm starting from 1 cm below the cecum) were immediately fixed with 10% paraformaldehyde for histological examination or temporarily stored in liquid nitrogen and then transferred to −80°C for transcriptome analysis.

All experimental procedures were approved by the Animal Experimentation Ethics Committee of Zhejiang University (No. 2021927). In addition, all animal experiments complied with the ARRIVE guidelines and the Guide for the Care and Use of Laboratory Animals of the National Research Council.

### Liver function tests

The serum levels of total protein, ALT, AST, alkaline phosphatase, cholinesterase, TBA, TBil, DBil, and GPDA were measured using a Hitachi 7600–210 automatic analyzer (Hitachi, Tokyo, Japan).

### Histological examination

The left lobes of the liver and ileum were fixed with 10% formaldehyde for 24 hours and embedded in paraffin. The tissue was cut into 2 µm sections and stained with HE. Liver tissue damage and intestinal mucosal lesions were assessed according to a previous study (20, 45). Tissue damage was blindly assessed using at least three slides from each specimen.

### Cytokine detection

The levels of the following cytokines in serum were analyzed using the Bioplex Pro Mouse Cytokine 23-plex Assay Kit (Bio Rad, Hercules, CA, USA): interleukin (IL)−1α, IL-1β, IL-2, IL-3, IL-4, IL-5, IL-6, IL-9, IL-10, IL-12 (p40), IL-12 (p70), IL-13, IL-17, eotaxin, granulocyte colony-stimulating factor (G-CSF), granulocyte-macrophage colony-stimulating factor (GM-CFS), interferon-γ (IFN-γ), chemokine ligand 1 (CXCL-1), monocyte chemoattractant protein 1 (MCP-1), macrophage inflammatory proteins (MIPs), including MIP-1α and MIP-3α, regulated upon activation, normal T-cell expressed and secreted (RANTES), and tumor necrosis factor-α (TNF-α).

### Detection of LPS and glutathione

Serum LPS and hepatic glutathione were detected using commercial ELISA kits (Jianchen, Nanjing, China).

### Gut microbiota analysis

DNA was extracted from 0.2 g of stool using the QIAamp DNA Stool Kit (Qiagen, Valencia, CA, USA). The universal primers 341F: 5′-CCTACGGGNGGCWGCAG-3′ and 805R: 5′-GACTACHVGGGTATCTAATCC-3′ were used to amplify the V3–V4 variable regions of 16S rDNA. PCR was performed in a 25 µL PCR system containing 50 ng of template DNA, 25 pmol of each primer, and 12.5 µL of Phusion Hot Start Flex 2× Master Mix (New England Biolabs, Ipswich, MA, USA). The PCR procedure was as follows: denaturation at 98°C for 30 s, 32 amplification cycles (denaturation at 98°C for 10 s, annealing at 54°C for 30 s, and extension at 72°C for 45 s), and extension at 72°C for 10 min. The PCR products were detected by 2% agarose gel electrophoresis and recycled using AMPure XT Beads (Agencourt, Beckman Coulter, USA). Sequencing was performed with an Illumina NovaSeq (Illumina, San Diego, CA, USA).

FLASH (v1.2.8) (46) was used to splice double-ended sequences. Fqtrim (v0.94) and Vsearch (v2.3.4) (47) were used to perform quality and chimeric filtering from the spliced fragments to obtain clean data. The relative abundance of each sample was normalized by the SILVA (release 132) classifier. The observed species, Chao1, Shannon index, and β-diversity were calculated with QIIME2 (v1.8.0). BLAST (https://blast.ncbi.nlm.nih.gov/Blast.cgi) was used for sequence alignment, and the annotation of each representative sequence was performed using the SILVA database (https://www.arb-silva.de/).

### Gut metabolome analysis

Gut metabolomics samples were prepared and analyzed in accordance with our previous method (9, 11). Briefly, 0.1 g of feces was mixed with 800 mL of ice-cold methanol for extraction; the extract mixture was then homogenized, centrifuged, and filtered, and the supernatant was subsequently vacuum freeze-dried, methoxymated, and trimethylsilylated with 20 mL of heptadecanoic acid (1 mg/mL) as an internal standard. The metabolites were assayed by gas chromatography‒mass spectrometry (GC‒MS) using an Agilent 7890A GC system coupled to an Agilent 5975C inert mass selective detector system (Agilent Technologies, Santa Clara, CA, USA).

Metabolites were first identified using the NIST databases (https://www.nist.gov/; score >80). OPLS-DA and VIP calculations were performed using SIMCA software (version 14.1; Sartorius Stedim Biotech, Umeå, Sweden).

### Transcriptome analysis of the liver and ileum

Total RNA extraction and purification were performed using TRIzol Reagent (Invitrogen, Carlsbad, CA, USA) and an RNA 1,000 Nano LabChip Kit (Agilent, Santa Clara, CA, USA). The RNA with an RNA integrity number ≥6.0 was enriched using oligo (dT) magnetic beads and reverse transcribed to cDNA using the NEBNext Ultra RNA Library Prep Kit for Illumina (New England Biolabs, Ipswich, MA, USA). Sequencing was performed with the Illumina HiSeq 4000 (Illumina, San Diego, CA, USA) platform.

Clean data were obtained from the raw data after removing reads containing adapters, reads containing N bases, and low-quality reads. The clean reads were compared with mouse reference genomes from UCSC (http://genome.ucsc.edu/) using HISAT2 (v2.0.5). StringTie (v1.3.3b) and HISAT2 (v2.0.5) were used for splicing fragments. FeatureCounts (v1.5.0-p3) was used to determine the level of mRNA expression by calculating each million mapped reads.

DESeq2 (v1.20.0) was used for differential expression analysis. The method described by Benjamini and Hochberg was used to adjust the *P* value. ClusterProfiler (v3.4.4) was used to analyze the statistical enrichment of differentially expressed genes in the KEGG pathway.

### Proteomics analysis of the liver

Total proteins were extracted from 50 mg of samples as described previously (9, 48). Peptides were labeled with TMT and analyzed using TMT Mass Tagging Kits and Reagents (Thermo, Rockford, IL, USA). Peptide fractionation and LC‒MS/MS for proteomics analyses were then also performed according to a previous study (48).

The resulting spectra were matched in the NCBI nr (https://www.ncbi.nlm.nih.gov/), UniProt (http://www.uniprot.org/), and BioGRID (https://thebiogrid.org/) databases using the search engine Proteome Discoverer 2.4 (PD 2.4, Thermo, Rockford, IL, USA) with the following search parameters: mass tolerance for precursor ions, 10 ppm; and mass tolerance for product ions, 0.02 Da.

The differential expression analysis of the two groups was performed with the DESeq2 R package (version 1.20.0). The *P* values controlled the false discovery rate under Benjamini and Hochberg’s approach. Genes belonging to different groups in the Venn diagram were plotted using the VennDiagram R package (version 1.6.20), and the enrichment of DEGs in KEGG pathways was calculated and plotted using the R package of clusterProfiler (version 3.5.1) and ggplot2 (version 3.1.1).

### RT-qPCR analysis

Total RNA of the liver was reverse transcribed into cDNA and measured by RT–qPCR with Premix Ex Taq (TaKaRa Biomedicals, Kusatsu, Japan) in the ViiA7 Real-time PCR system (Applied Biosystems, Waltham, MA, USA). The primer sequences for the target genes and the housekeeping gene glyceraldehyde-3-phosphate dehydrogenase (GAPDH) are provided in Supporting Table S1.

### Data analysis

The data were analyzed using SPSS 22.0 software (SPSS, Inc., Chicago, IL, USA), and the normality of the distribution was first tested using the Kolmogorov–Smirnov test. Then, one-way Analysis of Variance (ANOVA) with Bonferroni post hoc correction (for data with normal distributions) and the Kruskal–Wallis test (for data with nonnormal distributions) were used to assess the differences among groups. The adjusted *P* (*P*_adj_) value and Spearman correlations were analyzed using the fdrtool (v1.2.16) and corrplot packages (v0.84) of R, respectively.

## Data Availability

The datasets generated for this study can be found in Jianguoyun, the GenBank Sequence Read Archive Repository under accession number PRJNA801714 and ProteomeXchange Consortium under accession number PXD044099.

## SUPPLEMENTAL MATERIAL

Supplemental material msphere.00672-23-s0001.docx Tables S1 to S3 and Fig. S1.

ASM does not own the copyrights to Supplemental Material that may be linked to, or accessed through, an article. The authors have granted ASM a non-exclusive, world-wide license to publish the Supplemental Material files. Please contact the corresponding author directly for reuse.
