# Supplementary material for: The oral administration of Lacticaseibacillus casei Shirota alleviates acetaminophen-induced liver injury through accelerated acetaminophen metabolism via the liver-gut axis in mice
Source: mSphere. 2024 Jan 9;9(1):e00672-23. doi: 10.1128/msphere.00672-23 (PMC10826347; doi:10.1128/msphere.00672-23)
Supplement: Supplemental material — Tables S1 to S3 and Fig. S1. [file msphere.00672-23-s0001.docx]

The oral administration of *Lacticaseibacillus casei* Shirota alleviates acetaminophen-induced liver injury through accelerated acetaminophen metabolism via the liver-gut axis in mice

**Longxian Lv^1,3†^, Siqi Ren ^2†^, Huiyong Jiang^1,3†^, Ren Yan^1,3†^, Wenyi Chen^4^, Ruiyi Yan^4^, Jinming Dong^4^, Li Shao^5*^, Ying Yu^2*^**

^1^ State Key Laboratory for Diagnosis and Treatment of Infectious Diseases, Collaborative Innovation Center for Diagnosis and Treatment of Infectious Diseases, The First Affiliated Hospital, College of Medicine, Zhejiang University, Hangzhou 310003, Zhejiang, China

^2^ Key Laboratory of Biomarkers and In Vitro Diagnosis Translation of Zhejiang Province, School of Public Health, Hangzhou Medical College, Hangzhou 310063, Zhejiang, China

^3^ Jinan Microecological Biomedicine Shandong Laboratory, Jinan 250117, Shandong, China

^4^ School of Clinical Medicine, Hangzhou Medical College, Hangzhou 311300, Zhejiang, China

^5^ The Affiliated Hospital of Hangzhou Normal University, Institute of Translational Medicine, Hangzhou Normal University, Hangzhou 311121, Zhejiang, China

*** Correspondence:**Ying Yu, PhD, School of Public Health, Hangzhou Medical College, 182 Tianmushan Road, Hangzhou 310063, Zhejiang, China

Tel: 86-571-88215560

Email: [yuying@hmc.edu.cn](mailto:yuying@hmc.edu.cn)

Li Shao, PhD, The Affiliated Hospital of Hangzhou Normal University, Institute of Translational Medicine, Hangzhou Normal University, Hangzhou 311121, Zhejiang, China

Tel.: 86-571-87236453

Email: [shaolyly@zju.edu.cn](mailto:shaolyly@zju.edu.cn)

^†^ These authors contributed equally to this work.

Supplementary Table S1. RT‒qPCR primers used in this study

|  | Primers | Amplicon size (bp) | Tm (°C) | PrimerBank ID |
| --- | --- | --- | --- | --- |
| *Ugt2b34* | 5’-TGAAGTGATGGTTCTGAGACCT-3’ | 161 | 60 | 23956406a1 |
|  | 5’-ACTGCTTTGGCAGCTCATAAAT-3’ |  |  |  |
| *Ugt2b36* | 5’-CTACAAGTGGCTTCCCCAAAA-3’ | 97 | 60 | 71274183c1 |
|  | 5’-ATGGATCGCCTCGTAGAGTCC-3’ |  |  |  |
| *Gstt1* | 5’-CCGTCGCGCCATTTATATCTT-3’ | 122 | 60 | 31982317a1 |
|  | 5’-CCCTCTTCATGGGGTTCACC-3’ |  |  |  |
| *Gstp1* | 5’-ATGCCACCATACACCATTGTC-3’ | 161 | 60 | 10092608a1 |
|  | 5’-GGGAGCTGCCCATACAGAC-3’ |  |  |  |
| *Mgst1* | 5’-CTCAGGCAGCTCATGGACAAT-3’ | 110 | 60 | 31981068a1 |
|  | 5’-GTTATCCTCTGGAATGCGGTC-3’ |  |  |  |
| *Anpep* | 5’-ATGGAAGGAGGCGTCAAGAAA-3’ | 180 | 60 | 26252164a1 |
|  | 5’-CGGATAGGGCTTGGACTCTTT-3’ |  |  |  |
| *Idh1* | 5’-ATGCAAGGAGATGAAATGACACG-3’ | 116 | 60 | 6754278a1 |
|  | 5’-GCATCACGATTCTCTATGCCTAA-3’ |  |  |  |
| *Nat8* | 5’-ATGGCTTCTTTTCGCATCCG-3’ | 100 | 60 | 12963681a1 |
|  | 5’-GGAAGGCAGTGGGTATGTGC-3’ |  |  |  |
| *GAPDH* | 5’-AGGTCGGTGTGAACGGATTTG-3’ | 123 | 60 | 6679937a1 |
|  | 5’-TGTAGACCATGTAGTTGAGGTCA-3’ |  |  |  |

Supplementary Table S2. Pretreatment with LcS decreases APAP-induced ALI

|  | Ctrl | APAP | LcS | LcS + APAP |
| --- | --- | --- | --- | --- |
| Total protein (g/L) | 54.72 ± 1.74 | 43.52 ± 1.48 *** | 52.98 ± 3.51 | 44.40 ± 1.91 |
| ALT (IU/L) | 23.2 ± 1.87 | 13548.00 ± 2051.49 *** | 26.60 ± 14.73 | 13059.20 ± 2811.72 |
| AST (IU/L) | 84.00 ± 12.65 | 13130.40 ± 1857.45 *** | 93.80 ± 13.94 | 10126.40 ± 2607.05 ^###^ |
| GPDA (IU/L) | 52.00 ± 1.79 | 105.60 ± 3.92 *** | 51.76 ± 4.27 | 100.80 ± 12.74 |
| Alkaline phosphatase (IU/L) | 96.80 ± 8.39 | 136.00 ± 7.45 ** | 97.20 ± 7.13 | 144.80 ± 13.77 |
| Cholinesterase (IU/L) | 3788.00 ± 131.85 | 2389.60 ± 117.81*** | 3504.40 ± 113.14 | 2371.20 ± 151.26 |
| TBA (μmol/L) | 9.84 ± 0.24 | 62.80 ± 9.58 *** | 9.24 ± 0.29 | 43.36 ± 21.52 ^#^ |
| TBil (μmol/L) | 4.00 ± 1.11 | 10.08 ± 1.23 *** | 4.72 ± 0.96 | 7.44 ± 0.76 ^#^ |
| DBil (μmol/L) | 0.96 ± 0.63 | 2.40 ± 1.51 * | 0.64 ± 0.74 | 1.96 ± 1.11 |
| IBil (μmol/L) | 3.04 ± 1.00 | 8.00 ± 0.96 *** | 4.08 ± 1.01 | 5.48 ± 0.58 ^#^ |

Data are shown as the mean ± SEM; **p* < 0.05, ** *p* < 0.01, *** *p* < 0.001, when the APAP group or LcS group was compared to the Ctrl group; ^#^*p* < 0.05, ^###^ *p* < 0.001, when the LcS + APAP group was compared to the APAP group.

Supplementary Table S3. Pretreatment with LcS decreases the APAP-induced abnormal release of cytokines

|  | Ctrl | APAP | LcS | LcS + APAP |
| --- | --- | --- | --- | --- |
| Eotaxin (pg/mL) | 889.21 ± 76.37 | 564.52 ± 43.90** | 739.31 ± 66.09 | 1094.55 ± 115.31^###^ |
| G-CSF (pg/mL) | 1046.74 ± 245.22 | 2390.74 ± 431.47* | 1131.39 ± 309.23 | 2219.89 ± 722.86 |
| GM-CSF (pg/mL) | 19.30 ± 4.10 | 10.01 ± 4.91 | 13.20 ± 5.09 | 10.43 ± 10.36480 |
| IFN-γ (pg/mL) | 24.09 ± 2.93 | 11.20 ± 1.74*** | 21.34 ± 1.63 | 12.95 ± 1.76 |
| IL-1α (pg/mL) | 7.19 ± 0.75 | 16.88 ± 2.81*** | 7.20 ± 0.94 | 11.30 ± 2.09^#^ |
| IL-1β (pg/mL) | 2.80 ± 0.20 | 2.29 ± 0.35 | 2.31 ± 1.00 | 1.94 ± 0.41 |
| IL-2 (pg/mL) | 4.51 ± 1.02 | 7.49 ± 1.72 | 2.28 ± 0.52 | 8.64 ± 3.64 |
| IL-3 (pg/mL) | 23.71 ± 0.53 | 7.86 ± 1.26*** | 18.53 ± 3.38 | 6.86 ± 1.12 |
| IL-4 (pg/mL) | 0.85 ± 0.26 | 0.02 ± 0.02 | 0.65 ± 0.23 | 0.70 ± 0.20^#^ |
| IL-5 (pg/mL) | 5.23 ± 0.63 | 4.60 ± 0.89 | 7.22 ± 2.23 | 5.11 ± 0.36 |
| IL-6 (pg/mL) | 8.46 ± 3.08 | 38.54 ± 6.73** | 14.30 ± 4.03 | 32.43 ± 9.09 |
| IL-9 (pg/mL) | 28.89 ± 5.10 | 17.70 ± 2.70 | 23.49 ± 25.29 | 20.78 ± 4.72 |
| IL-10 (pg/mL) | 25.33 ± 1.63 | 40.72 ± 10.84 | 12.33 ± 2.10 | 27.88 ± 9.88 |
| IL-12(p40) (pg/mL) | 1362.67 ± 134.50 | 1358.90 ± 83.43 | 1029.85 ± 85.19 | 1274.43 ± 105.24 |
| IL-12(p70) (pg/mL) | 63.39 ± 8.58 | 4.40 ± 2.23*** | 45.68 ± 8.54 | 15.12 ± 7.09 |
| IL-13 (pg/mL) | 136.54 ± 41.77 | 61.93 ± 7.89* | 79.76 ± 10.05 | 55.83 ± 6.36 |
| IL-17α (pg/mL) | 133.22 ± 6.12 | 23.93 ± 5.09*** | 103.03 ± 10.95 | 26.37 ± 5.43 |
| CXCL-1 (pg/mL) | 60.75 ± 5.75 | 100.55 ± 12.42* | 54.98 ± 4.63 | 104.17 ± 15.33 |
| MCP1 (pg/mL) | 177.83 ± 20.68 | 757.07 ± 88.63*** | 191.54 ± 78.62 | 908.14 ± 62.65 |
| MIP1α (pg/mL) | 2.34 ± 0.24 | 1.49 ± 0.20* | 1.93 ± 0.29 | 1.76 ± 0.42 |
| MIP1β (pg/mL) | 190.86 ± 114.34 | 21.28 ± 17.40 | 136.28 ± 111.76 | 86.98 ± 35.09 |
| RANTES (pg/mL) | 259.38 ± 4.97 | 294.44 ± 24.45 | 197.20 ± 14.49 | 393.49±54.20 |
| TNF-α (pg/mL) | 82.92 ± 3.51 | 41.51 ± 13.36*** | 62.39 ± 8.87 | 30.56 ± 3.71 |

Data are shown as the mean ± SEM; **p* < 0.05, ** *p* < 0.01, *** *p* < 0.001, compared to the Ctrl group; ^#^*p* < 0.05, ^###^ *p* < 0.001, compared to the APAP group.


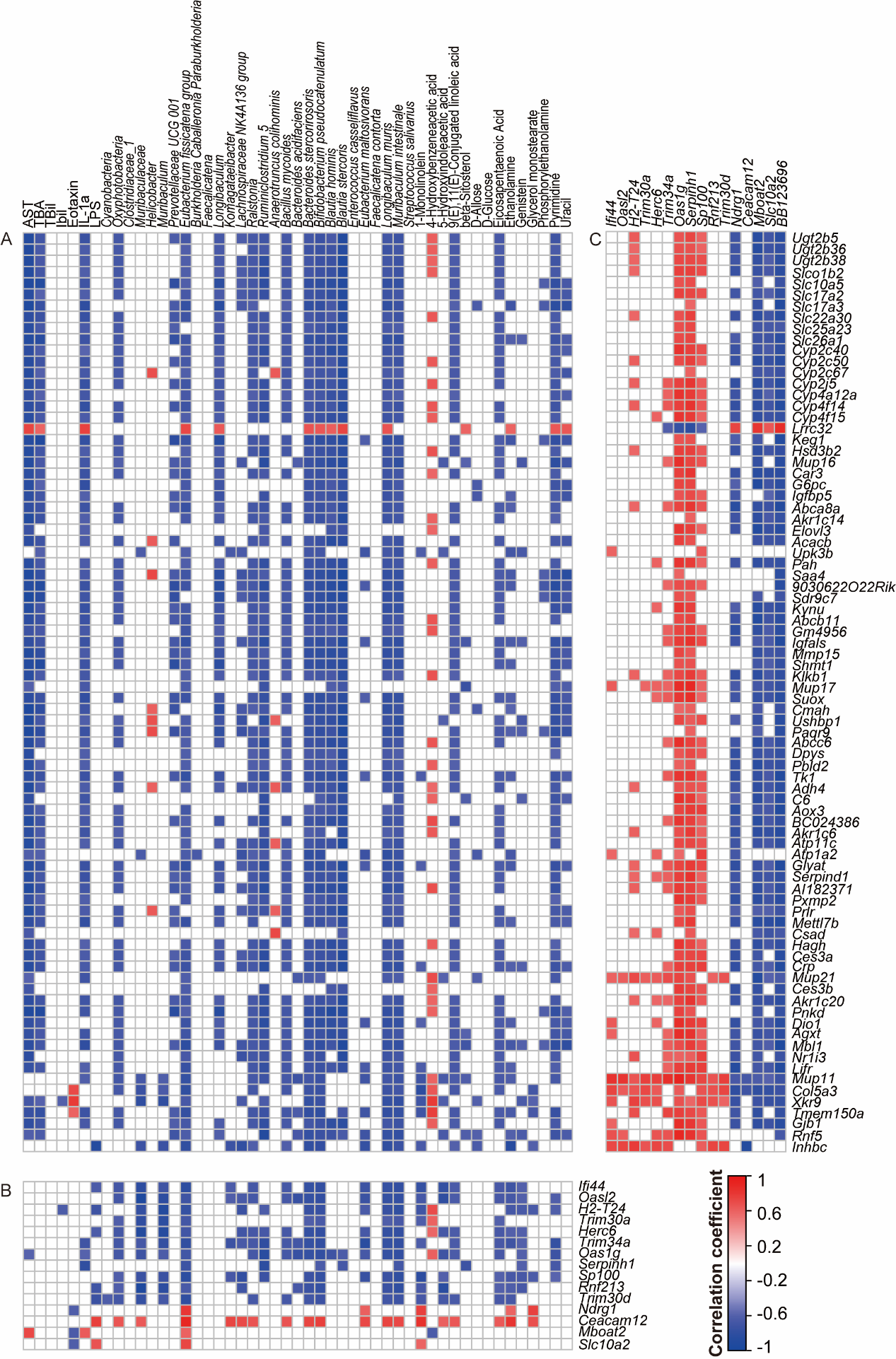


Supplementary Figure S1. More Spearman’s rank correlations between the APAP-induced LcS-alleviated indicators. (A) The correlations among the APAP-induced LcS-alleviated gut microbes, metabolites with liver function indicators, inflammatory cytokines, serum LPS and altered genes in liver. (B) The correlations among the APAP-induced LcS-alleviated gut microbes, metabolites with liver function indicators, inflammatory cytokines, serum LPS and altered genes in the ileum. (C) The correlations between liver proteins and ileum genes. The results with *P* values < 0.05 are shown in the heatmap. The color key and circle size indicate the strength of the correlation (r value). The red color indicates positive correlations; the blue color indicates negative correlations.
